# Sexual vulnerability of migrant women in the multicultural context of French Guiana: A societal issue

**DOI:** 10.3389/fpubh.2022.934049

**Published:** 2022-09-07

**Authors:** Leslie Alcouffe, Florence Huber, Pierre-Marie Creton, Luana Bitan, Adriana Gonzalez, Muriel Volpellier, Biancaelena Panfili, Antoine Adenis, Nicolas Vignier

**Affiliations:** ^1^Institute of Public Health Epidemiology and Development (ISPED), Bordeaux University, Bordeaux, France; ^2^French Red Cross, Saint Laurent du Maroni, French Guiana, France; ^3^Centre d'Investigation Clinique des Antilles et de la Guyane, CIC1424, Centre Hospitalier de Cayenne, Cayenne, French Guiana, France; ^4^Sorbonne Université, INSERM UMR 1136, Institut Pierre Louis d'Épidémiologie et de Santé Publique, IPLESP, Department of Social Epidemiology, Paris, France; ^5^French Collaborative Institute on Migration, Institut Convergences Migrations, ICM, Aubervilliers, France; ^6^Université Sorbonne Paris Nord, UFR SMBH, IAME, INSERM UMR 1137, Hôpital Avicenne, Hôpitaux Universitaires Paris Seine-Saint-Denis, AP-HP, Department of Infectious and Tropical diseases, Bobigny, France

**Keywords:** sexual health, sexual vulnerability, violence, transactional sex, migrants, women, French Guiana

## Abstract

**Background:**

French Guiana is a multicultural French territory in Amazonia with an old migration history and a high prevalence of HIV infection. The objective of this study was to evaluate situations of sexual vulnerability and their associated factors among migrant women in French Guiana.

**Methods:**

A cross-sectional epidemiological study was carried out in 2021 in the French Red Cross Prevention and Health Centers of the two main cities of French Guiana (Cayenne and Saint Laurent du Maroni). Analysis was performed with multivariate stepwise logistic regression using Stata 15.0 software.

**Findings:**

A total of 382 migrant women were included, with a median age of 31 years, mainly born in Haiti (80%), Suriname (9%), or Dominican Republic (6%), undocumented (71%), and with financial difficulties (77%). Among the 20% having casual partners, 57% reported unprotected sexual intercourse, more often the Haitian and Surinamese women. A history of rape was reported by 20% of women, most often in the country of origin (71%). Lifetime rape was associated with being threatened [aOR = 3.69 (1.96–6.96)] or being physically abused [aOR = 12.95 (6.51–25.75)] in the multivariate analysis. Among the women surveyed, 30% reported having ever exchanged sex for money, food, or shelter in their lifetime. Transactional sex is more common among Dominican women [aOR = 5.59 (1.84–16.95)] and women living in French Guiana for more than 2 years [aOR = 2.32 (1.38–3.92)]. Transactional sex is also associated with alcohol misuse [aOR = 2.57 (1.46–4.53)], history of threats [aOR = 2.03 (1.14–3.63)], history of rape [aOR = 1.92 (1.03–3.60)], and depressive disorders [aOR = 2.08 (1.21–3.60)].

**Interpretation:**

Migrant women in French Guiana are in a situation of sexual vulnerability. An intervention focused on sexual education and the promotion of prevention tools among Haitian women is advisable. Better prevention and support for transactional sex are needed to prevent violence and its mental health and alcohol misuse consequences for all women, especially Dominican women.

## Introduction

French Guiana is a French overseas territory that is located in the Amazon. It is the second largest region in France, but also the one with the lowest population density, that is, with 294,071 residents in 2021 (1). Migration is important and the demography is dynamic. The Guianese population is young (one out of two persons is under 25 years old) and mixed, with 38% of the census population being foreign-born nationals (2). The region is affected by precariousness, with more than 50% of the population living below the poverty line in 2017 and an unemployment rate of 23% in the same year (2). This precariousness particularly affects the migrants, with 74% of households being poor compared to 39% of those born in French Guiana (2). This precariousness is also expressed in access to healthcare; in 2016, 30.9% of Guianese declared that they had given up medical care for financial reasons over the past 12 months (3).

French Guiana is experiencing a generalized HIV epidemic. It is the French department with the highest HIV prevalence, that is, over 1% in 2016 (95% CI: 1.18–1.35%), with predominantly heterosexual transmission (4). Foreign-born individuals are overrepresented among people living with HIV in French Guiana, comprising more than 75% of the patient cohort. According to a study conducted on the basis of the slope of the CD4 count of patients followed for HIV in French Guiana, the majority of individuals have acquired HIV infection after the migration (5).

Concurrent sex, non-use of condoms, transactional sex, and sexual violence are considered to be the risk factors for HIV acquisition (6–8). Several studies, including the French ANRS Parcours study, report a greater sexual vulnerability with migration (9). Sexuality at risk of HIV acquisition was described among the migrant population in French Guiana during the 2012 KAPB survey. Of the respondents, 35.6% had engaged in at-risk sexual intercourse in the past 12 months (10). Regarding arrivals, women were identified to be particularly at risk of violence and more likely than men to use transactional sex (10, 11). The risk of contracting HIV is higher among women who have experienced nonconsensual sex after arrival in France (12). The link between transactional sex, multi-partnering, and HIV acquisition has also been established (13). Beyond practices, being a woman is a factor associated with a lack of awareness of the risk of exposure to STIs and with barriers to access to healthcare (3, 10).

Migrant women are described as being particularly vulnerable in terms of sexual health after their arrival in French Guiana. We hypothesize that newcomer migrant women are exposed to sexual violence, likely to engage in transactional sex, and have low condom use with their casual partners in relation to their living conditions. We also hypothesize that sexual behavior differs according to the country of origin and that women engaged in transactional sex have also experienced violence in their lives.

In this context, to evaluate situations of sexual vulnerability and their associated factors among migrant women, we conducted a survey to better understand the predisposing factors of sexual vulnerability among migrant women attending the French Red Cross Health and Prevention Centers (HPC) of the two main cities of French Guiana (Cayenne and Saint Laurent du Maroni).

## Materials and methods

The French Guiana Sexual and Reproductive Health of Migrant Women (*GuyaSSeReMig*) survey is a bicentric, descriptive, and analytical cross-sectional epidemiological study carried out in French Guiana in 2021 by French Red Cross and the Kikiwi health network in partnership with the clinical research center INSERM CIC 1424.

### Inclusion criteria

A migrant was defined according to the National Institute of Statistics (INSEE) and the High Council for Integration as a person who is born abroad, of foreign nationality, and residing in a French territory, including in the French overseas territories, regardless of their current nationality. Foreign-born women aged 18 years old and above who visited Health and Prevention Centers (HPCs) of the French Red Cross Prevention in Saint Laurent du Maroni and Cayenne between May and August 2021 were eligible to be included in the study.

### Data collection

The HPCs of the French Red Cross in French Guiana are medical and social structures involved in migrants' care. These structures carry out medical appointments for newcomers, screening for STIs, tuberculosis, vaccination and are the gateway to healthcare for people without health insurance coverage. They are places of choice to get in touch with newcomer migrants in French Guiana. These centers are used by the majority of first-time migrants, whether documented or undocumented, because they wish to be vaccinated with the mandatory yellow fever vaccine. Overall, around 15,000 people who visit the HPC yearly, among whom three-fourths are migrants.

Data were collected during 45-min face-to-face interviews conducted by trained interviewers in French, Haitian Creole, English, Portuguese, Spanish, or Sranan Tongo. The interview was administered by computer-assisted personal interviews (CAPI) and included 135 questions.

We used the conceptual framework for action on the social determinants of health of the WHO Commission on Social Determinants of Health to define the variable of interest. The covered themes were as follows: sociodemographic characteristics, administrative status, perceived health status, alcohol and drug use, pregnancy and childbearing history, sexual health and behavior, reproductive health, history of physical or sexual violence, and transactional sex. Following the interview, hygiene and prevention kits containing sanitary pads, soap, a toothbrush, toothpaste, masks, condoms, and lube were given to patients as a thank you gesture for the time spent on the survey. Mental health was assessed using the PHQ4 score, and alcohol consumption was assessed using the AUDIT-C score (14, 15).

### Sampling

Recruitment was done randomly at the end of the consultations, after the user has been informed by the healthcare worker.

The required number of participants was previously calculated as 350 to have sufficient power to identify the factors associated with sexual violence and transactional sex.

### Data analysis

Based on literature and a qualitative study conducted with healthcare workers, three outcomes were used to describe sexual vulnerability: not having used condoms with casual partners in the last 12 months, having been raped during the lifetime, and history of transactional sex (declaring having ever exchanged sex for food, money, or accommodation in the course of their life). These criteria were retained after the analysis of the literature as not permitting or making it difficult to negotiate protected sex and exposing persons in the Guianese context to the risk of acquiring HIV and other STIs. The selection of potentially associated variables was based on the purpose of the study, consistency with data from the literature conducted on the nearby populations, results of the qualitative study, and plausibility.

The data collected were described and analyzed using Stata® 15.0 software. The categorical variables were described in percentages and their associations using the Chi-square tests or Fisher's exact test. The quantitative variables were described by their mean or median values and studied using the Student's *t*-test.

The factors associated with the variables of interest were identified using Chi-square tests at the 0.20 *p*-value threshold. Then the variables were selected by manual top-down stepwise logistic regression by successively removing variables that were not significant at the 0.05 *p*-value threshold, starting with the variable with the largest *p*-value. At each stage of variable removal, the two models were compared using a likelihood ratio test. When the number of degrees of freedom allowed it, the adequacy of the models was verified by performing a Hosmer and Lemeshow test, and the discrimination capacity of the models was verified by measuring the area under the curve.

### Ethics and funding

All participants had given their informed consent, and information was collected anonymously. The GuyaSSeReMig survey that has been certified by the Data Protection Officer of the French Red Cross, in compliance with the reference methodology MR-003 of the French Data Protection Authority (CNIL), has been validated by the Sud-Méditerranée IV Protection of Persons Committee (Number 21.05.07.63207) and is registered in the National Agency for the Safety of Medicines and Health Products (ANSM) study register under the number ID-RCB 2021-A01311-40. While considering difficulties in participating in the survey due to poor or no knowledge of the French language, the information notice and the questionnaire have been translated into the native language of participants by the interviewers and interpreters.

This study was co-constructed in close collaboration with sexual health actors in French Guiana (regional HIV coordination (COREVIH), the Regional Health Agency (ARS), the French Red Cross, the AIDES and Doctors of the World NGOs, etc.). The study was funded by the Guianese Regional Health Agency with the institutional support of the French Red Cross, the Kikiwi network, and the Cayenne Hospital.

## Results

A total of 382 foreign-born women were surveyed. The median age was 31 years [IQR (25–37.5)]. The women were born mainly in Haiti (80.4%), Suriname (8.9%), and the Dominican Republic (5.7%). Among the respondents, 71.2% were undocumented at the time of the interview, 56.3% were housed, and 76.7% had a difficult or very difficult financial situation. Regarding the situation of the children, 13.1% send money to the children who stayed in their country of origin (Table 1).

**Table 1:** Descriptive sociodemographic data of migrant women over 18 years of age consulting the French Red Cross Health and Prevention Centers in Cayenne and Saint Laurent du Maroni in 2021 (*n* = 382).

	**Numbers and percentages**	
	***n***	**%**
**Age**	380	
Under 25 years old	112	29.5
26–35 years old	145	38.1
Over 35 years old	123	32.4
**Country of birth**	382	
Haiti	307	80.4
Dominican Republic	22	5.7
Surinam	34	8.9
Other countries	19	5.0
**Housing**	382	
Personal home	167	43.7
Hosted	215	56.3
**Year of arrival**	382	
Before 2020	211	55.2
2020 or 2021	171	44.8
**Level of education**	381	
Leaving school at or before secondary level	223	58.5
Higher to high school	158	41.5
**Current administrative status**	382	
Regular status	110	28.8
Undocumented	272	71.2
**Declared financial situation**	382	
Comfortable or very comfortable	24	6.3
Good	65	17.0
Difficult or very difficult	293	76.7
**Marital status**	307	
Single	195	63.5
Couple	44	14.3
Transnational couple	34	11.1
Married	31	10.1
Other	3	1.0
**Early pregnancy history**	382	
No early pregnancy	267	69.9
History of early ( ≤ 19 years old) pregnancy	115	30.1
**Children's situation**	382	
No children	130	34.0
Children without money transfer	202	52.9
Children with money transfer	50	13.1
**Alcohol consumption**	382	
Low consumption	293	76.7
Misuse	89	23.3
**Substance abuse (other drugs)**	382	
No	286	91.9
Yes	21	8.1
**History of rape**	382	
No	307	80.4
Yes	75	19.6
**History of transactional sex**	382	
No	269	70.4
Yes	113	29.6
**History of physical or partner violence**	382	
No	259	67.8
Yes	123	32.2
**Having been threatened during lifetime**	382	
No	281	73.3
Yes	101	26.7

According to the PHQ4 score, 57.6% of the women interviewed were screened with possible anxiety disorders and 60.7% of them with possible depressive disorders. In the sample, 23.3% of the women had alcohol misuse according to the AUDIT-C score.

Of the women surveyed, 19.6% had casual partners in the past 12 months, of which 57.3% had unprotected sex (Table 2).

**Table 2:** Univariate and multivariate analyses of factors associated with unprotected sexual intercourse with casual partners among migrant women attending Red Cross HPCs in 2021 using stepwise downward logistic regression (*n* = 75) AUC = 0.78.

	**Univariate analysis**			**Stepwise multivariate analysis**		
	**Crude OR**	**95%CI**	***p***	**Adjusted OR**	**95%CI**	***p***
**Country of birth**						
Haiti	**23.80**	**(4.61–122.79)**	**<0.001**	**23.80**	**(4.61–122.79)**	**<0.001**
Dominican Republic	1			1		
Surinam	**14.00**	**(1.84–106.46)**	**0.011**	**14.00**	**(1.84–106.46)**	**0.011**
Other countries	1.40	(0.10–19.01)	0.800	1.40	(0.10–19.01)	0.800
**Age**						
Under 25 years old	**4.40**	**(1.13–17.07)**	**0.032**			
26–35 years old	1.63	(0.53–5.04)	0.394			
Over 35 years old	1					
**Level of education**						
Leaving school at or before secondary level	**2.94**	**(1.11–7.80)**	**0.026**			
Higher to high school	1					
**History of transactional sex**						
No	1					
Yes	0,48	(0.16–1.43)	0.186			

Women from the Dominican Republic were identified by healthcare providers as being more informed than others about sexual health in the preliminary qualitative study and were chosen as the category of reference. Being born in Haiti or Surinam was associated with having unprotected sex with casual partners. No association was found between having unprotected sexual intercourse with casual partners and administrative status, family situation, housing, alcohol or drug use, perceived risk of STIs, consent at first intercourse, having suffered physical violence, pregnancy desire and the age difference between partners in a multivariate analysis.

Among the 382 migrant women, 19.6% reported having been raped in their lifetime, 70.7% of whom were in their country of origin (Table 3). The frequency of rape history was thus 13.9% in their country of origin and 4.2% in French Guiana.

**Table 3:** Univariate and multivariate analyses of factors associated with a history of rape among migrant women attending Red Cross HPCs in 2021, using stepwise logistic regression (*n* = 382) AUC = 0.85.

	**Univariate analysis**			**Stepwise multivariate analysis** **Extended model**		
	**Unadjusted OR**	**95%CI**	***p***	**Adjusted OR**	**95%CI**	***p***
**Country of birth**						
Haiti	1					
Dominican Republic	**3.32**	**(1.35–8.16)**	**0.009**			
Surinam	1.73	(0.76–3.91)	0.191			
Other countries	1.28	(0.41–4.00)	0.674			
**Early pregnancy History**						
No early pregnancy	1					
History of early ( ≤ 19 years old) pregnancy	**1.87**	**(1.11–3.16)**	**0.019**			
**Alcohol consumption**						
Low consumption	1					
Misuse	**2.06**	**(1.19–3.56)**	**0.010**			
**Substance abuse (other drugs)**						
No	1					
Yes	2.10	(0.94–4.66)	0.070			
**History of transactional sex**						
No	1					
Yes	**3.18**	**(1.88–5.36)**	**<0.001**			
**History of physical or partner violence**						
No	1			1		
Yes	**19.23**	**(9.94–37.23)**	**<0.001**	**12.95**	**(6.51–25.75)**	**<0.001**
**Having been threatened during lifetime**						
No	1			1		
Yes	**7.86**	**(4.53–13.67)**	**<0.001**	**3.69**	**(1.96–6.96)**	**<0.001**
**Depressive disorders**						
Absence of depressive disorders	**1**					
Depressive disorders	1.48	(0.87–2.53)	0.152			

Having been threatened or abused was associated with having been raped in the lifetime in the multivariate analysis. In contrast, there was no increased association between the country of birth and alcohol consumption after adjustment for violence and threats history. There was no association with age, level of education, or anxiety disorders.

Of the respondents, 29.6% reported having accepted sex in return for money, food or accommodation in their lifetime (transactional sex) (Table 4). “Stealthing”, the history of nonconsensual removal of condoms during sex, was more frequent among women with a history of transactional sex [unadjusted OR = 4.95 (2.35–10.45), *p* < 0.001, data not shown].

**Table 4:** Univariate and multivariate analyses of factors associated with transactional sex during lifetime among migrant women attending Red Cross HPCs in 2021, using stepwise logistic regression (*n* = 382)—AUC = 0.77, the Hosmer and Lemeshow test, *p* = 0.118.

	**Univariate analysis**			**Stepwise multivariate analysis** **Extended model**		
	**Unadjusted OR**	**95%CI**	***p***	**Adjusted OR**	**95%CI**	***p***
**Country of birth**						
Haiti	1			1		
Dominican Republic	**8.11**	**(3.06–21.45)**	**<0.001**	**5.59**	**(1.84–16.95)**	**0.002**
Surinam	1.66	(0.78–3.50)	0.186	1.15	(0.48–2.75)	0.748
Other countries	**2.74**	**(1.07–6.99)**	**0.035**	2.12	(0.77–5.88)	0,147
**Year of arrival**						
Before 2020	**2.28**	**(1.43–3.63)**	**0.001**	**2.32**	**(1.38–3.92)**	**0.002**
2020 or 2021	1			1		
**Early pregnancy history**						
No early pregnancy	1					
History of early ( ≤ 19 years old) pregnancy	**1.99**	**(1.25–3.17)**	**0.004**			
Children's situation			0.010			
**No children**	1					
Children but no money sent to them	1.20	(0.73–1.99)	0.470			
Children and sending money to them	**2.83**	**(1.43–5.60)**	**0.003**			
**Alcohol consumption**						
No or low consumption	1			1		
Misuse	**3.61**	**(2.19–5.93)**	**<0.001**	**2.57**	**(1.46–4.53)**	**0.001**
**Substance abuse (others drugs)**						
No	1					
Yes	**2.42**	**(1.15–5.08)**	**0.020**			
**History of physical or partner violence**						
No	1					
Yes	**2.80**	**(1.77–4.44)**	**<0.001**			
**Having been threatened during lifetime**						
No	1			1		
Yes	**2.84**	**(1.76–4.58)**	**<0.001**	**2.03**	**(1.14–3.63)**	**0.017**
**History of rape during lifetime**						
No	1			1		
Yes	**3.18**	**(1.88–5.36)**	**<0.001**	**1.92**	**(1.03–3.60)**	**0.041**
**Anxiety disorders**						
Absence of anxiety disorders	**1**					
Anxiety disorders	1.44	(0.91–2.26)	0.117			
**Depressive disorders**						
Absence of depressive disorders	1			1		
Depressive disorders	**1.98**	**(1.23–3.18)**	**0.005**	**2.08**	**(1.21–3.60)**	**0.008**
**History of incarceration**						
No previous incarceration	1					
Previous incarceration	**2.79**	**(1.30–5.99)**	**0.009**			

In multivariate analysis, being born in the Dominican Republic, being arrived before 2020, alcohol misuse, history of lifetime rape, threats and depressive disorders were independently associated with the history of transactional sex. The association of transactional sex with a history of pregnancy, children's status, drug abuse, history of physical violence, and history of incarceration were no longer significant once the previous factors were taken into account. No associations were found with the place of recruitment, age, level of education, number of dependent children, consent to first sexual intercourse, and anxiety disorder.

## Discussion

This original study on the sexual health of migrant women in French Guiana highlights the health needs of this vulnerable population.

The study reports low condom use in short-term relationships. Haitian and Surinam-born women are particularly vulnerable to unprotected sex with casual partners. Low condom use was first reported in 2012 among the migrant population in French Guiana, where 17% of men and 28% of women had reported not using a condom during the last intercourse with their casual partner (10). In contrast to this study, we did not find an association between these behaviors and age at first sexual intercourse. A study conducted in French Guiana among sex workers showed the importance of cultural and community aspects in the ability to negotiate condom use with one's intimate partner, while also highlighting the effectiveness of specific prevention and empowerment (16).

Exposure to violence and migration is a largely documented phenomenon in the literature. Women are particularly, though not exclusively, exposed to sexual violence and have little legal protection (17–19). The women in our sample are exposed to a significant level of sexual violence regardless of age, social level, or origin. Women who have been threatened or physically abused are also more likely to have been raped during their lifetime, thus underlining the close relationship between these different forms of violence.

In the general population in continental France, according to the Baromètre Santé survey conducted in 2016, 1 woman in 10 declared having experienced nonconsensual sexual intercourse in the course of her life (20). In the particular social context of French Guiana, the exposure is two times as high.

Most of the rapes reported by the respondents were experienced in the country of origin but continue to a lesser extent in French Guiana despite the recent arrival of the women studied. Exposure to sexual violence affects one woman in eight in Haiti, and this violence seems to be socially tolerated (21). This should remind us of the importance of implementing prevention, care and legal assistance measures in high-income territories.

Higher use of transactional sex among migrant men than in the general population has been described in French Guiana, with increased vulnerability among women (10). A 2012 study of migrants recruited in French Guiana informal housing areas reported that 8% of women declared having exchanged sex for money or drugs in their lifetime. In the current study, more than one in four women reported having exchanged sex for money, food or accommodation. This situation, although already reported among Dominican women, is non-marginal among all the migrants studied (22). Arriving before 2020 is a risk factor, suggesting an increase in risk over time in the context of survival or a different probability to experience cumulative incidence. However, the changing economic and migration context from 2020 onward tempers this statement. The profile of migrant women may also have changed during the COVID crisis. Transactional sex is also strongly associated with physical violence and threats, highlighting the cumulative and structural vulnerability identified in many studies (22, 23). From this point of view, all these hardships should be an alarm to better detect and accompany vulnerability. A study conducted in 2016 in the Democratic Republic of Congo by Doctors of the World reminds us that sexual behavior is part of a continuum, motivated as much by emotional reasons as by survival constraints. High-risk sexual behavior is closely linked to underlying sexual and gender-based violence and to gender relations of domination. This study also reminds us that one of the gaps in sex education is the prevention of sexual violence and protection from its consequences (24).

According to the HPC healthcare providers, sex workers are particularly exposed to nonconsensual condom removal (stealthing). This variable was associated with transactional sex. This phenomenon deserves to be known by healthcare professionals and social workers to improve the information for women, especially regarding HIV post-exposure prophylaxis as an emergency measure and the illegal nature of these practices.

Among people living with HIV in French Guiana, more than three-quarters were born abroad, and more than half of them became infected after arriving in the country. The vulnerability highlighted here is consistent with these elements and provides a better understanding of the factors driving this epidemic. Migrants are identified as particularly vulnerable victims of health inequities (25). Moreover, the conditions of access to services are also crucial in the Guianese context where the renunciation of care is particularly important (3). A 2015 study conducted in French Guiana, on the renunciation of care, suggested that vulnerable individuals had previous negative encounters with the healthcare system. Haitian people appeared to renounce more frequently. After multivariate analyses in this study, nationality was no more significant, suggesting that socioeconomic determinants were the main factors driving this association (26).

At last, the violence experienced and degraded mental health have an impact on the whole health of the people and require specific care and training of the caregivers, which are also the subject of reflection. For example, the reimbursement of psychologists' consultations and the establishment of psychologists in NGOs are currently being tested in French Guiana.

This survey has a certain number of limitations. First, some languages, such as Arabic, were not spoken by the interviewers, resulting in the exclusion of Syrian asylum seekers. However, the main countries of origin were well-represented with an adapted interpreting offer. The recruitment was done within the French Red Cross HPCs. The sample studied does not correspond to a random sample within the general migrant population of French Guiana. However, the women were recruited from the prevention and care centers of the French Red Cross. These centers are used by the majority of first-time migrants, whether documented or undocumented, because they wish to be vaccinated with the mandatory yellow fever vaccine. In addition, recruitment was carried out randomly within these health centers (recruitment of iterative consultants on the days when the interviewers were present). Thus, in view of the high frequency of use of these centers and the random recruitment, we believe that our sample is, nevertheless, a representative of the population of first-time migrant women in French Guiana. The sample may misrepresent the women furthest from the healthcare system. Given the topics covered, a prevarication bias is assumed. Finally, this is a cross-sectional survey, with a descriptive view at a given moment, which does not allow for a causal interpretation. This study should be used as a first step for future etiological studies. A biographical study (Parcours d'Haiti) is currently underway to better understand the temporal relationships between social status and sexual vulnerability among Haitian women in French Guiana.

This survey also has several strengths. The interviews were conducted in the women's language by interviewers trained in sexual health and active listening. Acceptability was very good, with many participants asking to return to speak with the interviewers again. The climate of trust established during the interviews encouraged people to express themselves and allowed them to be referred to healthcare providers when needed. This effect, described as “giving back a social existence to the participants”, in a process of empowerment, was also reported as an unexpected positive effect of the ongoing Makasi project conducted in the Paris region with migrants from Sub-Saharan Africa (27).

These elements remind us of the importance of systematically investigating the history of violence during the first health assessment and during all the appointments offered in the HPCs. Although much has been explored in key populations, women's needs, and experiences with STI screening, information regarding condom use and HIV pre-exposition prophylaxis (PrEP) is poorly documented and deserves to be investigated. A study conducted in America among women of different ethnic origins shows that women are highly interested in PrEP after receiving information (28). These elements remind us of the importance of information and the use of diversified prevention tools, particularly in the context of vulnerability. Psychological care must be significantly reinforced, with a systematic assessment of mental health during first-time consultations. Legal aid, considering the linguistic barrier, must be offered so that women can take legal action if they are exposed to violence. Women evolve in a very precarious situation and are socially invisible and vulnerable. The synergic action of the various actors and structures is necessary.

## Conclusion

This survey conducted in French Guiana on the sexual health of migrant women highlights the importance of taking care of women as a whole. Highly exposed to violence, transactional sex and non-protected sex, migrant women in French Guiana accumulate factors of sexual vulnerability. Systematic screening for violence and the extensive use of diversified prevention tools are key elements of risk reduction. Special attention should be given to Haitian-born migrant women and women reporting sexual transactions to inform and refer them to PrEP according to the French and international guidelines (28). Information and the implication of community actors play a major role in empowering women in terms of health, in addition to protecting them. The determinants of health concern individual characteristics, the living environment, the social system, and the global context. Thus, long-term and sustainable responses require intervention at multiple levels and inevitably rely on the protection of human rights in French Guiana. To reach women who reside far from the healthcare system, a mobile study in informal housing areas would be recommended. A survey more specific to the sexual behaviors of Haitians with a biographical collection of events, as well as a more specific survey of Dominican sex workers, would be useful.

## Data availability statement

The raw data supporting the conclusions of this article will be made available by the authors, without undue reservation.

## Ethics statement

The studies involving human participants were reviewed and approved by Comité de Protection des Personnes IDF-7. Written informed consent for participation was not required for this study in accordance with the national legislation and the institutional requirements.

## Author contributions

LA participated in writing the protocol, implemented the field study, coordinated the project, conducted interviews, and analyzed the data. FH supervised the writing of the protocol, supervised the study, secured funding, and analyzed the data. She was the principal investigator of the survey. PMC participated in writing, fundraising, and overseeing the implementation of the study. LB participated in the writing, conducted interviews, and analyzed the data. AG participated in the implementation of the survey. MV and BP participated in the construction of the project and implementation of the survey. AA supervised the writing of this article. NV supervised all the work of writing the protocol, questionnaires, and analyses. All authors participated in the writing and proofreading of this manuscript.

## Funding

The research was funded by Regional Health Agency of French Guiana and Institutional support from the French Red Cross and Kikiwi network.

## Conflict of interest

The authors declare that the research was conducted in the absence of any commercial or financial relationships that could be construed as a potential conflict of interest.

## Publisher's note

All claims expressed in this article are solely those of the authors and do not necessarily represent those of their affiliated organizations, or those of the publisher, the editors and the reviewers. Any product that may be evaluated in this article, or claim that may be made by its manufacturer, is not guaranteed or endorsed by the publisher.
